# UTILIZATION OF 4MS TRAINING MODEL IN PRIMARY CARE SETTINGS WITH SOCIAL WORK LEARNERS

**DOI:** 10.1093/geroni/igae098.2167

**Published:** 2024-12-31

**Authors:** Anna Faul, Samantha Cotton, Pamela Yankeelov

**Affiliations:** University of Louisville, Louisville, Kentucky, United States; University of Louisville, Louisville, Kentucky, United States; University of Louisvlle, Louisville, Kentucky, United States

## Abstract

The UofL GWEP used our FlourishCare curriculum (FCC) to train 52 social work (SW) learners on the 4Ms of age-friendly care. The FCC provides a variety of learning experiences, eg. online modules, workshops, interprofessional case management experiences, case conceptualization meetings, and Project ECHO’s. The FCC was delivered during the two-semester SW internship. The experiential part of the FCC allowed learners to work with small caseloads of patients incorporating the 4Ms in assessment, engagement and care navigation. Learners received weekly supervision from clinical SWers trained in the 4M model. The Kirkpatrick model of evaluation was used to evaluate the FCC, focusing on learner satisfaction, knowledge gain, self-efficacy to practice the 4M model, and patient outcomes. Evaluation data shows 95% of learners were satisfied/very satisfied with the learning experience. Learners showed a significant gain in knowledge related to the 4Ms of age-friendly care (Pre-M=65, SD=7.2; Post-M=76, SD=8.1). Learners significantly improved in in their self-efficacy to perform 4M age-friendly care (Pre-M=5.2, SD=1.3; Post-M=8.3,SD=1.9). Patient outcomes data, as measured by our biopsychosocial FlourishCare Index assessment showed that patients served by these learners significantly improved in their overall Index scores (Pre-M=50,SD=7.9; Post-M=59,SD=6.2), their psychological health (Pre-M=52,SD=5.4;Post-M=67,SD=6.1), and their health behaviors (Pre-M=49,SD=7.6; Post-M=58,SD=6.5). These results show the effectiveness of the FCC to develop a future SW workforce who has the ability to provide 4M age-friendly care.

